# Psychobiological Factors of Sexual Functioning in Aging Women – Findings From the Women 40+ Healthy Aging Study

**DOI:** 10.3389/fpsyg.2019.00546

**Published:** 2019-03-13

**Authors:** Laura Mernone, Serena Fiacco, Ulrike Ehlert

**Affiliations:** ^1^Department of Clinical Psychology and Psychotherapy, Institute of Psychology, University of Zurich, Zurich, Switzerland; ^2^University Research Priority Program Dynamics of Healthy Aging, University of Zurich, Zurich, Switzerland

**Keywords:** sexual functioning, female sexuality, women’s health, midlife and older age, biopsychosocial factors

## Abstract

**Background:** A variety of biological and psychosocial factors are associated with women’s sexual health in midlife and older age. Evidence suggests a decline in sexual functioning in the context of aging and the menopausal transition, including changes in sexual desire, arousal, lubrication, orgasm, pain, and/or contentment. However, not all women in midlife and older age experience such a decline, and it remains unclear how the endocrine environment and psychosocial aspects contribute to the maintenance of healthy sexual functioning. Therefore, the aim of this study was to examine psychobiological predictors of sexual functioning in healthy middle-aged and elderly females.

**Methods:** A total of 93 healthy, sexually active women aged 40–73 years completed a battery of validated psychosocial questionnaires, including measures of sexual functioning (Female Sexual Function Index) and of protective psychological traits and interpersonal variables. The steroid hormones estrogen, testosterone, progesterone and dehydroepiandrosterone sulfate were determined in saliva samples, while follicle-stimulating hormone, luteinizing hormone and sex hormone-binding globulin were determined in dried blood spots. The findings were statistically adjusted for multiple testing.

**Results:** Age and postmenopausal status were negatively associated with overall sexual functioning, arousal, and lubrication. Regression analyses revealed that relationship satisfaction, emotional support, self-esteem, optimism, and life satisfaction each significantly predicted overall sexual functioning or specific aspects of sexual functioning, including arousal, contentment, orgasm, and pain (all *p* < 0.029). For desire and lubrication, no associations were found with the tested psychosocial factors. In terms of steroid hormones, testosterone was positively linked to orgasm (*p* = 0.012). In this sample, 79.6% reported to have healthy sexual functioning according to the questionnaires’ cutoff. Younger age (OR = 0.911, 95% CI 0.854–0.970, *p* = 0.004) and a higher level of emotional support (OR = 1.376, 95% CI 1.033–1.833, *p* = 0.029) were associated with the presence of healthy sexual functioning.

**Discussion:** Although aging and menopause negatively affected aspects of sexual functioning, the accompanying endocrine correlates were not predictive for sexual functioning in this healthy sample of middle-aged and older females. Instead, our findings suggest that sexual functioning is highly dependent on psychosocial aspects related to well-being. Accordingly, personality traits such as optimism, and interpersonal aspects such as emotional support and relationship satisfaction were identified as important predictors of sexual functioning.

## Introduction

As populations are aging rapidly all over the world, there has been a growing interest in healthy aging and associated factors. One important aspect that is often overlooked or treated as taboo in aging populations is sexual health (Lusti-Narasimhana and Beard, 2013), even though sexuality is considered to be a fundamental part of being human and a central component of general health (Graugaard, 2017). According to the definition of the World Health Organization, sexual health refers to “a state of physical, emotional, mental, and social well-being in relation to sexuality, and not merely the absence of disease, dysfunction, or infirmity” (World Health Organization, 2006, p. 5). There is considerable knowledge concerning the positive association of sexual health with general well-being and health-related quality of life (Davison et al., 2009; Lee et al., 2016; Greenberg et al., 2017).

Historically, it was assumed that sexuality and its associated physical response does not differ between men and women (Masters and Johnson, 1966). Accordingly, physical sexual response is characterized by the linear progression from excitement, to plateau, orgasm, and finally resolution. However, this classical model was frequently criticized due to its sole focus on the physical (genital) response and was therefore extended with the aspect of sexual desire preceding the excitement phase (Kaplan, 1979). Despite this adaptation, it has been debated whether this linear model of sexual response adequately reflects women’s sexual experience. Basson (2001) proposed an alternative circular model, which specifically refers to the female’s sexual response. This model emphasizes the importance of emotional intimacy (as a result of a satisfying sexual experience) for future willingness to engage in sexual activity. According to the fifth edition of the Diagnostic and Statistical Manual of Mental Disorders (DSM-5), sexual functioning encompasses a complex interplay of biological, psychological, and sociocultural factors and can be significantly disturbed when a person’s ability to respond sexually or experience sexual pleasure is impaired (American Psychiatric Association, 2013).

Women’s sexual functioning can vary, or permanently change, across the life course, especially in the context of reproductive events and aging (Clayton and Harsh, 2016). There is a growing body of research investigating sexual functioning in middle-aged women experiencing the menopausal transition. Most of these studies have found a decline in sexual functioning in association with the menopausal transition (Dennerstein et al., 2003; Avis et al., 2017), with hormonal changes and associated vaginal dryness assumed to be the key factors in this decline. Moreover, studies have also looked at the aging process independently of menopausal status. Sexual functioning is thought to decline with increasing age. This may be due to an overall decline in general health as women age, physical changes or medical conditions (Clayton and Harsh, 2016). The age-associated decline in sexual functioning has been underpinned by prevalence studies of sexual dysfunction in middle and older age. For example, Nicolosi et al. (2004) assessed prevalence rates of sexual dysfunction in approximately 14,000 women aged 40–80 years from 29 countries. More than 65% of all women reported to have engaged in sexual activity in the past year. Among these, 38% reported having sexual intercourse more than once a week. The most prevalent sexual dysfunction in middle-aged and elderly women was a lack of sexual interest (21%), followed by inability to reach orgasm (16%) and lubrication difficulties (16%), no pleasure during intercourse (15%), and pain during intercourse (10%). A clear age pattern was detected, with increased prevalence rates with higher age (Nicolosi et al., 2004). According to an elaborated overview of the worldwide prevalence of sexual dysfunction provided by McCabe et al. (2016), the prevalence rates for low levels of female sexual desire vary between 17 and 55%. Negative age effects were observed after the age of 60, when the prevalence rates mostly range between 40 and 50%. Arousal difficulties have often been operationalized as lubrication insufficiencies, with varying prevalence rates of 21 to 28%. The prevalence of orgasmic dysfunction varies between 16 and 37%. Pain during sexual activity seems to be less prevalent in the general population, ranging from 1 to 27% across various studies (McCabe et al., 2016).

In sum, sexual dysfunction seems to increase in midlife and older age, including changes in sexual desire, arousal, lubrication, orgasm, pain, and/or contentment. However, there is a high numerical variability across different studies and countries, and there are some methodological issues which limit the generalizability of the findings (Hayes and Dennerstein, 2005). Furthermore, there is no consensus regarding the aspects of sexual functioning that change with advancing age. It is important to acknowledge, however, that not all women in midlife and older age experience a decline in sexual functioning. In a study by Trompeter et al. (2012), most of the surveyed sexually active women aged between 40 and 99 years reported frequent arousal, lubrication, and orgasm, even if sexual desire was low. Additionally, sexual satisfaction was higher in older women and was not associated with sexual activity. Lonnèe-Hoffmann et al. (2014) found similar results, demonstrating no significant changes in sexual functioning in women who maintained their sexual activity from early to late postmenopause. These and other studies reveal that sexual functioning does not necessarily worsen over the course of aging and that research to date has mainly focused on sexual dysfunction and negative aspects of sexuality among elderly women (Lusti-Narasimhana and Beard, 2013). So far, studies focusing on sexual health and positive aspects of sexual functioning are sparse. A focus on sexual function rather than dysfunction might be a more favorable approach in the investigation of females’ sexual health. Insights into factors associated with the maintenance of healthy sexual functioning could potentially be of considerable benefit in terms of positive health research.

**FIGURE 1:**
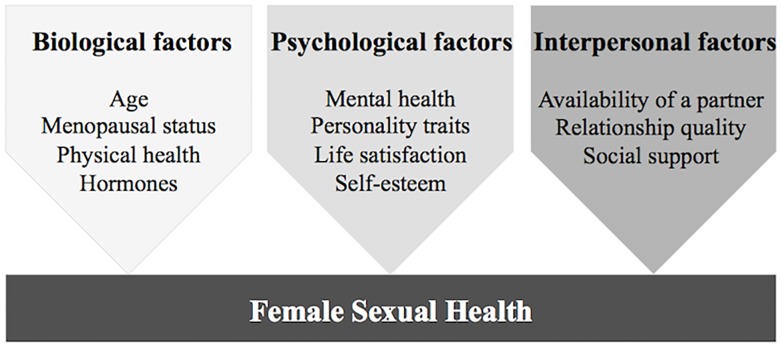
Biopsychosocial model of female sexual health in midlife and older age: biological, psychological, and interpersonal factors potentially contributing to healthy sexual functioning.

As reflected in the WHO definition of sexual health, which takes various dimensions into consideration and emphasizes the role of well-being (World Health Organization, 2006), sexual functioning should be defined positively and within a biopsychosocial framework (Graugaard, 2017). Such a perspective on sexual health in midlife and older age has been established in the past few years (Conklin, 2017). A central issue in the investigation of female sexual functioning is the importance of other factors besides menopausal status and age (e.g., Dennerstein et al., 2003). Psychosocial factors, particularly psychological and interpersonal variables, seem to play a crucial role in middle-aged and elderly women’s sexual functioning (DeLamater and Karraker, 2009; Thomas and Thurston, 2016). As presented in Figure 1, biological determinants include, among other factors, age, menopausal status, and hormonal changes related to menopause and aging (Fiacco et al., 2018). In particular, sex steroids can exert an impact on sexual functioning due to the fluctuations which occur with aging and in the context of the menopausal transition (Clayton and Harsh, 2016). Estradiol (E2) is the best-examined sex steroid with regard to sexual functioning in middle and older age. E2 levels show a sharp decline during the menopausal transition, causing vaginal atrophy, dryness and irritation, which may indirectly lead to decreased sexual desire, arousal, and response (Wierman et al., 2010). Moreover, androgens also seem to be associated with female sexual functioning. Testosterone (T) is the sex steroid which is assumed to primarily influence sexual desire and motivation (Bachmann and Leiblum, 2004; Wåhlin-Jacobsen et al., 2015). However, the role of estrogens and androgens in relation to female sexual functioning is highly controversial, since findings are inconsistent and many studies did not report any associations (Meston and Frohlich, 2000; Wierman et al., 2010). For dehydroepiandrosterone sulfate (DHEA-S), which is the most abundant sex steroid in females and an androgen precursor, mostly positive associations with sexual functioning have been reported (e.g., Davis et al., 2005). Little attention has been paid to progesterone (P), follicle-stimulating hormone (FSH), luteinizing hormone (LH), and sex hormone-binding globulin (SHBG) in relation to female sexual functioning (Dennerstein et al., 2005; Randolph et al., 2015; Worsley et al., 2016). To conclude, more research is required in order to further examine the contribution of sex steroids to women’s sexual functioning in midlife and older age, and non-hormonal factors also need to be taken into account.

As illustrated in Figure 1, psychological factors determining sexual functioning may include good mental health (absence of psychiatric conditions), specific psychological factors related to well-being such as self-esteem or life satisfaction, and protective personality traits. Psychiatric disorders constitute a risk factor for female sexual dysfunction (Basson and Gilks, 2018), with depression and anxiety in particular showing negative associations with sexual functioning (Althof et al., 2005). Therefore, the absence of mental disorders such as depression or anxiety may be protective for female sexual health. Another psychological factor related to subjective well-being that may have a positive impact on sexual functioning is general life satisfaction. To date, only a small number of studies have pointed out the relevance of life satisfaction for sexual functioning in women (Woloski-Wruble et al., 2010; Stephenson and Meston, 2015). Other studies have found a high level of self-esteem to be a positive psychological factor in female sexuality (Goodson et al., 2006; Rehbein-Narvaez et al., 2006). Additionally, personality traits seem to be significant for sexual health: A recent meta-analysis linking the Big Five personality traits (McCrae and John, 1992) to sexual health showed that a high level of extraversion was associated with fewer symptoms of sexual dysfunction and a higher frequency of sexual activity (Allen and Walter, 2018). Moreover, a higher degree of extraversion was also found to be related to greater sexual satisfaction (Allen and Desille, 2017) and to a higher level of orgasmic frequency and overall sexual functioning (Harris et al., 2008; Crisp et al., 2015).

Finally, besides biological and psychological determinants of sexual functioning in midlife and older age, interpersonal factors are crucial in the investigation of female sexual health (Figure 1). First, the availability of a partner is an important factor for the engagement in sexual activity and therefore sexual functioning (DeLamater and Karraker, 2009). If they have a partner, the majority of middle-aged and older women remain sexually active (Thomas et al., 2015). The general satisfaction with one’s partner or quality of communication in the relationship are crucial for females in order to experience satisfying sexuality (Byers, 2005; Thomas and Thurston, 2016). Social support from one’s partner may be another interpersonal factor which affects sexuality due to its health-promoting effects (Uchino, 2006), but this has not yet been investigated in the context of women’s aging. Finally, the partner’s physical or mental health status may have an impact on females’ sexual functioning (DeLamater and Karraker, 2009).

To summarize, it can be stated that first, female sexuality in midlife and older age has often been studied in terms of dysfunction and risk factors. Little attention has been given to factors that may predict female sexual health in midlife and older age. Hence, the present study focused on aspects of sexual functioning instead of dysfunction, and on associated protective factors. Furthermore, we investigated exclusively healthy women, since studies with a solely healthy female sample are scarce. Second, a biopsychosocial approach that simultaneously considers biological, psychological, and interpersonal factors should be applied when investigating female sexual functioning. The questions of what the most important determinants of sexual health in midlife and older age are, and how psychosocial aspects and the endocrine environment in these stages of life contribute to the maintenance of healthy sexual functioning, have not been investigated extensively. Therefore, the aim of this study was to examine psychobiological predictors of sexual functioning in a healthy sample of middle-aged and elderly females. For this purpose, we examined the associations of protective psychological and interpersonal factors, as well as endocrine factors (sex hormones), with female sexual functioning and its various components.

## Materials and Methods

This study was part of the Women 40+ Healthy Aging Study, a large research project that was conducted at the Department of Clinical Psychology and Psychotherapy of the University of Zurich. The goal of the research project was to investigate healthy middle-aged and older women using a biopsychosocial framework.

### Study Participants and Procedure

**FIGURE 2:**
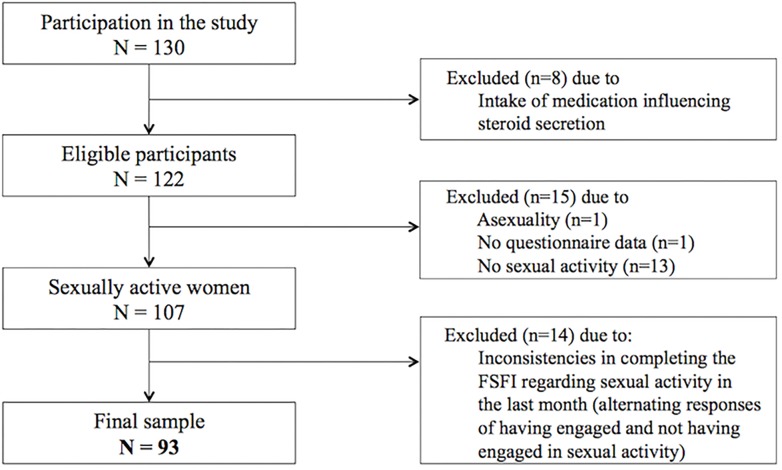
Participant flow chart.

In total, 130 self-reporting healthy women aged 40–73 years participated in the study. The sample was recruited among the general population using online advertisements and flyers. Participants’ self-reported health status was used as inclusion criteria. The participants had to report either a good, very good, or excellent health condition, and had to state that they were currently free of any acute or chronic somatic disease or mental disorder. Furthermore, none of the participants had received psychotherapy or psychopharmacological treatment in the previous 6 months. Additional exclusion criteria were applied regarding the assessment of hormones: pregnancy in the last 6 months; precocious menopause or menopausal status due to surgical removal of either both ovaries or the uterus; current use of oral contraceptives or use of hormone therapy in the last 6 months; any disease influencing the endocrine system; current diabetes mellitus, polycystic ovary syndrome, hirsutism, or endometriosis. Our study population included women with pre-, peri-, and postmenopausal status. According to the frequently used classification system *Stages of Reproductive Aging Workshop +10* (Harlow et al., 2012), we considered women with regular menstrual cycle as premenopausal; women with variable cycle length or an interval of amenorrhea of >60 days as perimenopausal; and women without menstrual bleeding in the last 12 months or more as postmenopausal. As illustrated in the participant flow chart (Figure 2), 37 women had to be excluded from the total sample for the data analyses. Most of these women reported not having had any sexual activity in the last month, which is a prerequisite for the appropriate use of the instrument measuring female sexual functioning (Rosen et al., 2000). Unfortunately, some women showed inconsistencies when reporting whether they engaged in sexual activity and therefore had to be excluded. Therefore, the total and final sample for the present analyses amounted to *N* = 93 healthy and sexually active women.

The study procedure consisted of two parts: In a first step, participants were invited to the laboratory for a physiological assessment, including the standardized collection of biological material for the analyses of endocrine parameters. Second, they completed validated psychosocial questionnaires online. In order to control for the menstrual cycle phase in premenopausal women, the physiological and psychosocial assessments were applied in the follicular phase. Written informed consent was obtained from all participants prior to data collection. The Cantonal Ethics Committee (KEK) of the canton of Zurich and the Cantonal Data Protection Commission of the canton of Zurich approved the study. The study was conducted in accordance with the principles of the Declaration of Helsinki.

### Psychosocial Measures

Psychosocial measures included protective psychological traits and interpersonal factors. All psychosocial measures were assessed online via self-reports using validated questionnaires. The participants were explicitly informed about the procedure and expected duration (approximately 30 min) of the online assessment prior to filling in the questionnaires. It was required to complete all questionnaires consecutively.

#### Sexual Functioning

Sexual functioning was examined using the German version of the Female Sexual Function Index (FSFI; Rosen et al., 2000) validated by Berner et al. (2004). The FSFI is a multidimensional self-report instrument and is considered to be the gold standard for the assessment of female sexual functioning. It contains 19 items covering six key domains of female sexual functioning: desire, arousal, lubrication, orgasm, satisfaction, and pain. Each domain comprises two to four items assessing the frequency, the level (degree) of, and/or satisfaction with the corresponding sexual functioning domain. Response options varied depending on the type of item. For items measuring frequency, the response options ranged from 0 (*no sexual activity*) to 5 (*almost always or always*). Items measuring the level of the specific sexual functioning domain or the satisfaction with it were either rated as 0 (*no sexual activity*) or rated from 1 (*very low or none at all/very dissatisfied*) to 5 (*very high/very satisfied*). To obtain individual domain scores for each sexual functioning domain, the scores of the corresponding items were added up and multiplied by the domain factor. Additionally, a full-scale score (total FSFI score) was calculated by summing up the six domain scores. Full-scale scores range from 2 to 36, with higher scores indicating better overall sexual functioning. According to Wiegel et al. (2005), women with and without sexual dysfunction can be discriminated by a total FSFI cutoff value of 26.55. Regarding the psychometric properties of the German version of the FSFI, Berner et al. (2004) reported satisfactory results in their validation study, with internal consistencies for the FSFI domains and total score ranging from α = 0.75 (satisfaction) to α = 0.95 (pain). In the present study, we found an excellent internal consistency for the total FSFI score (Cronbach’s alpha = 0.92) and a range from α = 0.74 (satisfaction) to α = 0.91 (pain) for the FSFI domains. The estimated duration to complete the FSFI is approximately 10 min (Berner et al., 2004).

#### Protective Psychological Markers

##### Self-esteem

The Multidimensional Self-Esteem Scale (MSES; Schütz and Sellin, 2006) was used to assess overall self-esteem. This self-report scale comprises a total of 32 items rated on a 7-point Likert scale, which examine six different aspects of self-esteem: emotional self-esteem, social skills, social confidence, achievement-related self-esteem, physical attractiveness, and sportiness. Furthermore, a global self-esteem score can be computed which includes all dimensions, and for which satisfactory reliability and validity (Cronbach’s alpha = 0.93) have been reported (Schütz and Sellin, 2006). This global score was used in the present study; internal consistency in the present study was acceptable (Cronbach’s alpha = 0.77). It takes approximately 10–15 min to complete the MSES (Schütz and Sellin, 2006).

##### Optimism

Dispositional optimism was measured with the German version of the Life Orientation Test-Revised (LOT-R) developed by Glaesmer et al. (2008). The LOT-R encompasses 10 items (three items each for optimism and pessimism, four neutral items) that are rated on a 5-point Likert scale. The item scores are added up to build an optimism and a pessimism scale. Only the optimism scale was used in this study. Glaesmer et al. (2008) reported an internal consistency of α = 0.69 for the optimism scale. Despite this rather questionable value, the authors argue that factor analysis clearly supports a two-factor structure and that the scale is applicable for research purposes. This is also supported by the short duration required to complete the LOT-R (approximately 5 min).

##### Extraversion

The short version of the Big Five Inventory (BFI-K; Rammstedt and John, 2005) was used to evaluate extraversion. The BFI-K contains 21 items rated on a 5-point Likert scale and depicts the Big Five personality traits: extraversion, neuroticism, conscientiousness, agreeableness, and openness. To build the trait subscales, the corresponding four to five item scores per trait are added together. In this study, only the extraversion subscale was used. Rammstedt and John (2005) reported satisfactory psychometric properties of the BFI-K, with an internal consistency ranging between α = 0.81 and 0.86 for the extraversion scale in different samples. The Cronbach’s alpha of extraversion in this study lay at 0.79, implying an acceptable internal consistency. The BFI-K is a particularly economic instrument, since the average duration to complete all items is under 2 min (Rammstedt and John, 2005).

##### Life satisfaction

Participants’ life satisfaction was measured with the most commonly used instrument in this area, the Satisfaction with Life Scale (SWLS; Glaesmer et al., 2011). The SWLS comprises five items rated on a 7-point Likert scale and is completed within few minutes. In a German validation study, Glaesmer et al. (2011) found very good internal consistency for the SWLS (Cronbach’s alpha = 0.92). Cronbach’s alpha in the present study lay at 0.87.

#### Interpersonal Factors

##### Relationship satisfaction

The Relationship Assessment Scale (RAS; Sander and Böcker, 1993) was used to evaluate participants’ satisfaction with their intimate relationship. The RAS is a brief measure, only comprising seven items rated on a 5-point Likert scale. Validation studies found satisfactory results regarding reliability and validity of the instrument (Sander and Böcker, 1993). In the present study, the internal consistency of the RAS was excellent, with a Cronbach’s alpha of 0.91.

##### Emotional support

Emotional support was assessed using the Berlin Social Support Scales (BSSS; Schwarzer and Schulz, 2000). The BSSS measures the level of emotional support, instrumental support, search for social support, and need for social support using 17 items rated on a 4-point Likert scale. Only the emotional support scale was used in this study. The validity and reliability of the BSSS have been demonstrated in several studies (Schulz and Schwarzer, 2003). In the present study, Cronbach’s alpha lay at 0.83 for the emotional support scale. Completion of the BSSS takes approximately 10 min.

### Endocrine Measures

Saliva samples as well as capillary blood samples were collected in order to analyze endocrine parameters. The steroid hormones estradiol (E2), testosterone (T), progesterone (P4), and DHEA-S were assessed in saliva. Measurement of these steroids in saliva samples is well-established and reliable (for review, see Lewis, 2006; Gröschl, 2008). FSH, LH, and SHBG were determined in dried blood spot (DBS) samples. DBS sampling is an eligible and valid method to quantify these biomarkers, since highly sensitive and precise assays exist for the determination of FSH, LH, and SHBG (Worthman and Stallings, 1997; Edelman et al., 2007; McDade et al., 2007). Furthermore, DBS sampling is a viable and effective alternative to blood sampling by venipuncture, because it is less invasive and expensive, as well as easier to collect and store (Edelman et al., 2007).

Both saliva and capillary blood samples were obtained under standardized conditions at 8.00 am in the laboratory of the Department of Clinical Psychology and Psychotherapy of the University of Zurich. Prior to the sampling, the participants answered questions about incidents (e.g., infection or cold in the last week) and activities (e.g., sleep difficulties and subjective stress level at that moment) that could potentially have biased the current hormone concentrations.

#### Saliva Sampling

Saliva was collected with a SaliCap sampling tube of 2 mL capacity (IBL International GmbH, Hamburg, Germany). Participants were instructed to let the saliva flow to the base of the mouth before drooling into the tube using a polypropylene straw (passive drool method). Following the collection, saliva samples were stored at -20°C. The samples were thawed and centrifuged prior to biochemical analysis using IBL Saliva Immunoassays (IBL International GmbH, Hamburg, Germany). All saliva samples were analyzed in the biochemical laboratory of the Department of Clinical Psychology and Psychotherapy at the University of Zurich.

#### Dried Blood Spot Sampling

For the assessment of steroid hormone concentrations in dried blood, small capillary blood samples were drawn from participants’ fingers. A sterile, disposable lancet (Accu-Chek^®^ Safe-T-Pro Plus) was used for the finger prick on the middle finger of the non-dominant hand. Capillary blood drops were spotted onto standardized filter paper (Whatman^®^ Protein Saver Cards, No. 903). The DBS samples were dried for 4 h and then frozen at -20°C. All DBS measures were analyzed in the Cytolab laboratory in Regensdorf, Switzerland.

### Statistical Analyses

Statistical analyses were conducted using the Statistical Package for the Social Sciences (SPSS, version 23). To compare pre- and postmenopausal women regarding their level of sexual functioning, a Mann-Whitney *U*-test (non-parametric alternative procedure to the independent samples *t*-test) was used in order to account for the heteroscedastic variances. In addition, we calculated a one-way ANOVA (Analysis of Variance) including age as a covariate, to examine whether differences in sexual functioning between pre- and postmenopausal women remain significant when adjusting for age. To examine the associations between main variables (age, psychosocial factors, and endocrine factors) and sexual functioning, bivariate and partial correlation analyses were calculated in a first step. To further determine the strength of these associations and to test possible predictors of sexual functioning, simple and multiple linear regression analyses were conducted in a second step. In addition, we examined the associations between the main variables and the dichotomous outcome of sexual functioning (absence vs. presence of healthy sexual functioning) according to the FSFI cutoff value of 26.55 proposed by Wiegel et al. (2005). For that reason, we first used bivariate correlation analyses to discover significant associations, and subsequently calculated logistic regression analyses to predict the odds of the dichotomous outcome. For all analyses testing the associations of psychosocial factors and sexual functioning, age and BMI were included as covariates. Additionally, systolic and diastolic blood pressure, medication intake and smoking status were statistically considered when testing the associations of steroid hormones and sexual functioning. Steroid hormone values were not normally distributed and were therefore log-transformed prior to the analyses. For all analyses including salivary steroids, 24 participants were excluded from the analyses due to gum bleeding (*N* = 2), an infection or injury in the mouth cavity (*N* = 3), or having had a cold (*N* = 19) in the days before the laboratory assessment. To adjust for multiple testing, we used the false discovery rate proposed by Benjamini and Hochberg (1995). The α-value of 0.05 was adjusted by (n+1)/2n, taking into account seven dependent variables per comparison (six FSFI domain scores and total score). Therefore, an α-value of *p* < 0.029 was considered statistically significant. For all analyses testing the associations of the main variables with the dichotomous outcome of sexual functioning (absence vs. presence of healthy sexual functioning), the level of statistical significance was set at α = 0.05.

## Results

### Sample Characteristics

The final sample consisted of *N* = 93 healthy middle-aged and elderly women aged 40–73 years. Table 1 provides the sociodemographic and health-related sample characteristics. The vast majority of participants were either pre- or postmenopausal. Regarding education, a vocational education or a college/university degree were most frequently reported. More than half of the sample was married, while the remaining participants were either single or in a common-law relationship. All of the participants were either hetero- or bisexual. About two thirds of the participants had their own children. The vast majority of the sample neither smoked nor took any medication.

**Table 1:** Descriptive statistics of sociodemographic and health-related sample characteristics (*N* = 93).

**Age, M ± SD**	52.5 ± 8.5
**Menopausal status, *n* (%)**	
Premenopausal	45 (48.4)
Perimenopausal	6 (6.5)
Postmenopausal	42 (45.2)
**Education, *n* (%)**	
Vocational education	39 (41.9)
High school-leaving certificate	11 (11.9)
College/university degree	37 (39.8)
Other	6 (6.5)
**Relationship status, *n* (%)**	
Single	10 (10.8)
In a relationship	29 (31.2)
Married	54 (58.1)
**Sexual orientation, *n* (%)**	
Heterosexual	88 (94.6)
Bisexual	5 (5.4)
**BMI (kg/m^2^), M ± SD**	22.71 ± 3.62
**Blood pressure (mmHg), M ± SD**	
Systolic blood pressure	121.77 ± 13.83
Diastolic blood pressure	79.58 ± 8.34
**Smoking, *n* (%)**	
Yes	12 (12.9)
No	81 (87.1)
**Medication, *n* (%)**	
Yes^∗^	8 (8.6)
No	85 (91.4)


*^∗^Reported medication included antihypertensive drugs, cholesterol-lowering drugs, dietary supplements or other (acid blockers and gastric protection)*.

### Sexual Function Characteristics

Descriptive statistics of sexual function characteristics (total FSFI and domain scores) are presented in Table 2. Higher scores in the FSFI domains indicate better sexual functioning. In our sample, 74 women (79.6%) scored above the cutoff value proposed by Wiegel et al. (2005), indicating healthy sexual functioning. As our sample comprised middle-aged and elderly women (Age: *M* = 52.5 years, *SD* = 8.5 years), we examined the impact of age and menopausal status on sexual functioning.

**Table 2:** Descriptive statistics of sexual function characteristics.

FSFI domain	*M* ±*SD*	*MIN, MAX*	*Range*
Desire	6.03 ± 1.36	2.00, 10.00	2–10
Arousal	15.96 ± 3.31	5.00, 20.00	0–20
Lubrication	16.82 ± 3.70	5.00, 20.00	0–20
Orgasm	12.45 ± 2.51	5.00, 15.00	0–15
Contentment	13.00 ± 2.28	5.00, 15.00	2–15
Pain	13.55 ± 2.45	2.00, 15.00	0–15
Total sexual functioning	29.05 ± 4.28	13.20, 36.00	2–36


*Data are presented as mean (*M*) ± standard deviation (*SD*), minimum (*MIN*), maximum (*MAX*) scores, and range. Higher scores in the FSFI domains indicate better sexual functioning*.

#### Age and Sexual Functioning

Age was negatively associated with arousal (*r* = -0.302, *p* = 0.003), lubrication (*r* = -0.401, *p* < 0.001), and overall sexual functioning (*r* = -0.283, *p* = 0.006). These negative associations were further confirmed using linear regression analyses. The results demonstrated that the lower the women’s age, the higher their overall sexual functioning (*β* = -0.267, *p* = 0.010, adj. *R^2^* = 0.61), arousal (*β* = -0.295, *p* = 0.004, adj. *R^2^* = 0.077), and lubrication (*β* = -0.393, *p* < 0.001, adj. *R^2^* = 0.145). Furthermore, age was negatively associated with having healthy sexual functioning (*r* = -0.318, *p* = 0.002). Binary logistic regression analysis revealed that age [*b* = -0.094, Wald(1) = 8.312, *p* = 0.004] was a significant predictor of having healthy sexual functioning [Chi-Square(1) = 9.206, *p* = 0.002]. The odds ratio (OR) for age was 0.911 (95% CI 0.854-0.970), which means that for one added year of life, the relative probability to have healthy sexual functioning decreases 8.9% in the sample. Nagelkerke’s *R*^2^ was 0.148.

#### Menopausal Status and Sexual Functioning

**FIGURE 3:**
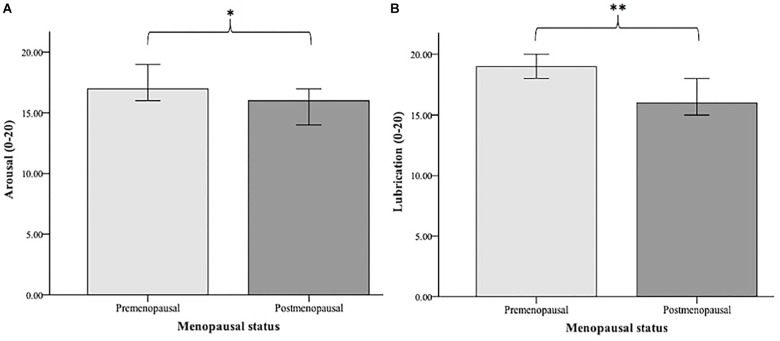
Median values of arousal **(A)** and lubrication **(B)** in premenopausal (*N* = 45) vs. postmenopausal women (*N* = 42). Arousal and lubrication were measured using the Female Sexual Function Index (FSFI; Rosen et al., 2000). Significance level: ^∗^*p* < 0.05, ^∗∗^*p* < 0.01.

Premenopausal (*N* = 45) and postmenopausal (*N* = 42) women were compared in terms of their sexual functioning. A Mann-Whitney *U*-test indicated that arousal was greater for premenopausal (*Mdn* = 17.00) than for postmenopausal (*Mdn* = 16.00) women (*U* = 693.000, *p* = 0.031). The two groups also differed significantly regarding lubrication (*U* = 608.000, *p* = 0.003), with premenopausal women having greater lubrication (*Mdn* = 19.00) than postmenopausal women (*Mdn* = 16.00). The significant group differences are illustrated in Figure 3.

In addition, we analyzed whether the differences in sexual functioning between pre- and postmenopausal women remained significant when adjusting for age. A one-way ANOVA including age as a covariate revealed that this was not the case.

### Associations Between Psychosocial Factors and Sexual Functioning

To investigate the contribution of protective psychosocial factors to sexual functioning, we examined relationship-related factors (relationship satisfaction and emotional support) and psychological factors (self-esteem, optimism, extraversion, and life satisfaction) as possible predictors. In a first step, partial correlations between these psychosocial factors and sexual functioning were calculated, as illustrated in Table 3. The associations were further analyzed using regression analyses. Controlling for age and BMI, linear regression analyses revealed that overall sexual functioning (total FSFI score) was significantly predicted by optimism (*β* = 0.247, *p* = 0.018, adj. *R^2^* = 0.136), relationship satisfaction (*β* = 0.307, *p* = 0.005, adj. *R^2^* = 0.122), and emotional support (*β* = 0.246, *p* = 0.015, adj. *R^2^* = 0.110). Life satisfaction, self-esteem and extraversion were not significant predictors of overall sexual functioning (all *p* > 0.029).

**Table 3:** Summary of the partial correlation coefficients for the association of psychosocial factors with sexual functioning (*N* = 93).

	RAS	BSSS-ES	MSWS	LOT-R-O	BFI-K-E	SWLS
Desire	0.255	-0.012	0.053	0.222	0.105	0.080
Arousal	**0.297^∗∗^**	0.209	0.201	**0.253^∗^**	0.102	0.135
Lubrication	0.124	0.011	–0.019	0.007	–0.002	–0.011
Orgasm	0.041	0.246	0.186	**0.274^∗∗^**	0.168	0.081
Contentment	**0.488^∗∗∗^**	**0.379^∗∗∗^**	0.278^∗∗^	0.223	0.218	**0.335^∗∗^**
Pain	0.227	**0.271^∗∗^**	0.243	0.125	0.158	0.116
Total sexual functioning	**0.314^∗∗^**	**0.254^∗∗^**	0.215	0.248^∗^	0.169	0.163


*Covariates: age, BMI. RAS, relationship satisfaction; BSSS-ES, emotional support; MSWS, self-esteem; LOTR-O, optimism; BFI-K-E, extraversion; SWLS, life satisfaction. Significance level: ^∗^*p* < 0.029 (adjusted for multiple testing), ^∗∗^*p* < 0.01, ^∗∗∗^*p* < 0.001*.

The associations between the psychosocial factors and sexual functioning were further examined regarding the FSFI domains desire, arousal, lubrication, orgasm, contentment, and pain, using regression analyses. Optimism (*β* = 0.251, *p* = 0.015, adj. *R^2^* = 0.120) and relationship satisfaction (*β* = 0.289, *p* = 0.007, adj. *R^2^* = 0.126) each predicted arousal. Optimism was also a significant predictor of orgasm (*β* = 0.282, *p* = 0.009, adj. *R^2^* = 0.064). Sexual contentment was significantly predicted by the following psychosocial factors: relationship satisfaction (*β* = 0.484, *p* < 0.001, adj. *R^2^* = 0.239), emotional support (*β* = 0.370, *p* < 0.001, adj. *R^2^* = 0.172), self-esteem (*β* = 0.272, *p* = 0.008, adj. *R^2^* = 0.107), and life satisfaction (*β* = 0.330, *p* < 0.001, adj. *R^2^* = 0.141). The domain pain was only predicted by emotional support (*β* = 0.271, *p* = 0.009, adj. *R^2^* = 0.056). None of the tested psychosocial factors were predictive for the domains desire and lubrication (all *p* > 0.029).

In addition, we examined the associations between the psychosocial factors and the dichotomous outcome of sexual functioning (absence vs. presence of healthy sexual functioning). Emotional support was positively associated with having healthy sexual functioning (*r* = 0.253, *p* = 0.015), adjusted for age and BMI. Binary logistic regression analysis indicated that emotional support [*b* = 0.319, Wald(1) = 4.759, *p* = 0.029] was a significant predictor of healthy sexual functioning [Chi-Square(3) = 15.447, *p* = 0.001]. The OR for emotional support was 1.376 (95 % CI 1.033 – 1.833), which means that as emotional support increases one unit, the relative probability to have healthy sexual functioning increases 37.6%. Nagelkerke’s *R^2^* was 0.240.

### Associations Between Endocrine Factors and Sexual Functioning

As illustrated in Table 4, partial correlation analysis revealed that overall sexual functioning was not associated with any of the tested steroid hormones (adjusted for age, BMI, systolic and diastolic blood pressure, medication, and smoking status). When investigating the single FSFI domains and steroid hormones, testosterone was positively associated with orgasm. Regression analyses further confirmed that testosterone was a significant predictor of orgasm (*β* = 0.329, *p* = 0.012, adj. *R^2^* = 0.089).

**Table 4:** Summary of the partial correlation coefficients for the associations of steroid hormones with sexual functioning (*N* = 93 for blood steroids, *N* = 69 for salivary steroids).

	E2	P4	T	DHEA-S	FSH	LH	SHBG
Desire	0.132	0.006	0.083	-0.233	-**0.235^†^**	-0.165	0.084
Arousal	0.055	0.085	**0.256^†^**	-0.069	-0.077	-0.141	-0.070
Lubrication	0.060	0.023	0.060	-0.158	-0.145	-0.129	0.056
Orgasm	0.057	0.193	**0.316^∗^**	0.033	0.084	0.046	-0.047
Contentment	-0.035	-0.019	0.012	-0.207	-0.091	-0.114	-0.046
Pain	-0.001	0.103	0.068	-0.084	-0.131	-0.111	-0.006
Total sexual functioning	0.057	0.089	0.176	-0.153	-0.116	-0.136	-0.008


*Covariates: age, BMI, systolic and diastolic blood pressure, medication, smoking status. For the salivary steroids, *N* = 24 participants were excluded due to reports of a cold, infection, or gum bleeding before the laboratory assessment. E2, estradiol; P4, progesterone; T, testosterone; DHEA-S, dehydroepiandrosterone sulfate; FSH, follicle-stimulating hormone; LH, luteinizing hormone; SHBG, sex hormone-binding globulin. Significance level: ^∗^*p* < 0.029 (adjusted for multiple testing), ^†^*p* < 0.05*.

Additionally, we examined the associations between the endocrine factors and the dichotomous outcome of sexual functioning (absence vs. presence of healthy sexual functioning). Partial correlation analysis (including the covariates) showed no significant associations with the tested steroid hormones. Therefore, the endocrine factors were not entered into a binary logistic regression model.

## Discussion

This study investigated psychobiological predictors of sexual functioning in a healthy sample of middle-aged and elderly females. For this purpose, we examined the associations of age, menopausal status, protective psychological and interpersonal factors, as well as endocrine factors with female sexual functioning and its various components. First, we found that age and menopausal status were negatively associated with the level of sexual functioning in the sample. Second, psychosocial factors emerged as the key predictors of sexual functioning. To our knowledge, this is the first study to test and demonstrate the importance of optimism and emotional support for healthy women’s sexual functioning in midlife and older age. Third, we found that sex hormones were not related to sexual functioning in this healthy sample.

As our sample comprised middle-aged and elderly women (40–73 years), it was necessary to take into account age and menopausal status for the investigation of sexual functioning. Negative effects of age on female sexual functioning are frequently reported in the literature and also reflected in the prevalence rates of sexual dysfunction across different age ranges (e.g., Nicolosi et al., 2004). However, the findings differ in terms of which aspects of sexual functioning are affected by age. In the present study, younger age was associated with higher levels of arousal, lubrication, and overall sexual functioning. This is partly consistent with the existing literature. Most of the previous studies investigated the effects of age on sexual desire, interest, and frequency of sexual activity (reviewed in Hayes and Dennerstein, 2005), with the majority of findings revealing a decrease in sexual desire and interest (e.g., DeLamater and Sill, 2005). Research on the effects of age on arousal in females is limited (Hayes and Dennerstein, 2005). The available studies have reported both a decline in arousal with increasing age (Lee et al., 2016) as well as no changes (Cain et al., 2003). The negative relationship between age and lubrication can be biologically explained by age-associated changes in the urogenital system. As women age, vaginal symptoms such as vaginal dryness emerge more often due to the reduced production of mucus from the glands of the vaginal wall as a sign of vaginal atrophy (Samsioe, 1998). However, these age-associated physiological changes are confounded by the hormonal effects of the menopausal transition phase, and therefore need to be carefully disentangled from the effect of menopausal status on sexual functioning (Dennerstein et al., 2001). In our study, however, we found that menopausal status was only related to sexual functioning independently of age. Finally, the high correlation between sexual arousal and lubrication (as one of the main physiological underpinnings of arousal) may explain why both of these aspects are associated with age.

Regarding the relevance of menopausal status for sexual functioning, we found that pre- and postmenopausal women only differed in their level of sexual arousal and lubrication. Overall sexual functioning differed only at a trend level (*p* < 0.10). These findings are only partly consistent with the literature. According to various longitudinal studies, the transition to menopause is related to some, but not necessarily all, aspects of sexual functioning. For example, Avis et al. (2000) found a lower level of sexual desire and arousal with advancing menopausal status, while other aspects of sexual functioning remained unrelated to menopausal status. In another study, lubrication emerged as the domain of sexual functioning with the most pronounced decline during the transition to menopause (Gracia et al., 2007). Other findings revealed that pain during sexual intercourse increases, while only sexual desire decreases across the menopausal transition (Avis et al., 2009). These inconsistencies might be due to methodological issues concerning the differing measurement of sexual functioning or the dependency of sexual functioning on other factors besides age and menopausal status, such as psychosocial factors in midlife and older age.

In the present study, we found several psychosocial predictors of sexual functioning. A high level of relationship satisfaction and emotional support were associated with better overall sexual functioning in this healthy sample. The importance of relationship-related factors for sexual health in midlife and older age has already been discussed in the literature (Brotto et al., 2016). In line with previous research findings, relationship satisfaction seems to be among the most important determinants of female sexual functioning (Dennerstein et al., 2005; Thomas and Thurston, 2016). Our results provide additional evidence for the importance of relationship satisfaction, because it was a predictor not only of sexual contentment, but also of arousal and overall sexual functioning. The partner plays a key role for a woman’s satisfying sexuality, since the partner’s sexual functioning may have an impact on the females’ sexual functioning (DeLamater and Karraker, 2009). There is evidence showing a strong correlation between females’ and males’ sexual functioning (Yeoh et al., 2014). Therefore, one’s own sexual wellbeing may not only be dependent on the satisfaction with the relationship, but also on the partner’s sexual wellbeing. However, social support, and in particular emotional support, had not been previously investigated. Interestingly, our study showed that emotional support was a predictor of overall sexual functioning as well as of particular aspects, namely sexual contentment and pain. Moreover, to our knowledge, the present study was the first to examine the role of optimism for the sexual functioning of healthy middle-aged and elderly females. Optimism is defined as a personality disposition characterized by generalized positive outcome expectancies, and has been shown to be positively associated with physical health and mental well-being (Carver and Scheier, 2014). We found that a higher level of optimism was related to a higher level of overall sexual functioning, arousal, and orgasm. Furthermore, life satisfaction and self-esteem were associated with sexual contentment in our study, but not with overall sexual functioning. We assume that life satisfaction may be specifically connected to satisfaction in other areas of life, such as sexuality, which would explain the association with sexual contentment. Self-esteem represents a central dimension of the self-concept, which has been shown to have health-promoting effects on a variety of outcomes regarding, for example, emotion, cognition, and motivation (Goodson et al., 2006). So far, the relationship between self-esteem and sexual functioning has not been investigated in detail. Our results are partly in line with a study showing that collegiate women with higher self-esteem reported greater sexual contentment and orgasmic response (Rehbein-Narvaez et al., 2006). However, there is a lack of studies including women in middle and older age. Finally, it is interesting to note that none of the tested psychosocial factors in this study were related to sexual desire or lubrication. Further research is needed to confirm our findings and to examine psychosocial factors associated with desire and lubrication.

As part of the biopsychosocial approach, we also examined the role of endocrine factors in the sexual functioning of healthy females. None of the tested endocrine parameters (E2, T, DHEAS, P, FSH, LH, and SHBG) were linked to overall sexual functioning. This argues against the assumption that sex steroids are important for female sexual functioning due to their age- and menopause-associated fluctuations (Clayton and Harsh, 2016). From that perspective, it is surprising that sex steroids were not associated with sexual functioning in our sample of pre- and postmenopausal women. From yet another perspective, however, our findings are in line with the majority of current studies and overviews pointing to weak or no correlations between sex steroids and female sexual functioning (Meston and Frohlich, 2000; Dennerstein et al., 2005; Wierman et al., 2010; Randolph et al., 2015; Worsley et al., 2016). An explanation for the observed results in this study may lie in the rather low variability across our study sample. All of our participants reported to be physically and mentally healthy, did not take any hormonal agents and reported rather low levels of menopausal symptoms, which may indicate an adaptive adjustment to hormonal fluctuations. Taking this into account, we can speculate that endocrine factors might only be determinants of sexual dysfunction rather than sexual function. Additional research is required to test this hypothesis and to further examine the contribution of sex steroids to women’s overall sexual functioning in midlife and older age. Another potential explanation for the non-significant findings may concern the validity of the saliva samples for the hormones measured in our elderly sample. Although the assessment of E2 in saliva samples is valid and salivary E2 is considered as an adequate estimate of serum E2 (Fiers et al., 2017), there is data suggesting that salivary E2 may only predict serum E2 among postmenopausal women who use estrogen therapy (Tivis et al., 2005). According to the authors, E2 levels may be too low among postmenopausal women who do not use estrogen therapy and therefore cannot be adequately detected via saliva samples.

However, we found that a specific aspect of sexual functioning, namely orgasm, was positively associated with the level of testosterone. Testosterone is the sex steroid which is assumed to primarily influence sexual desire and motivation (Bachmann and Leiblum, 2004; Wåhlin-Jacobsen et al., 2015). Evidence for the importance of testosterone in female sexual functioning mainly stems from studies demonstrating that testosterone replacement therapy improves sexual functioning (Shifren et al., 2000; Goldstat et al., 2003). Pharmacological agents such as testosterone or Flibanserin, a 5-hydroxytryptamine 5-HT1A agonist and 5-HT2A antagonist, are frequently reviewed as treatment options for poor sexual desire and low sexual functioning in mostly premenopausal women (Roslan et al., 2019; Simon et al., 2019). However, their efficacy and safety is the subject of controversial discussion in the current literature.

The present study has several strengths. First, it is one of the few studies to investigate exclusively healthy females in midlife and older age. Strict inclusion criteria ensured that the examined women were physically and mentally healthy, and did not take any hormone replacement or psychotropic medication. Second, we applied a biopsychosocial health approach and therefore included various perspectives, examining biological, psychological, and interpersonal factors of the complex phenomenon of female sexual functioning. Furthermore, we focused on positive and protective factors in relation to optimal sexual functioning, which have rarely been investigated so far. This notion is in line with positive health concepts that stress the importance of strengths and resources rather than risks and pathology (Graugaard, 2017).

Some limitations of the present study should also be acknowledged. The Women 40+ Healthy Aging Study has a cross-sectional design, in which psychosocial and hormonal data were gathered at only one time point. Therefore, it was not possible to infer causality of the tested associations. Further, larger studies are required to confirm our results. Additionally, future longitudinal examinations are needed to examine changes in sexual health and functioning over time and the associated psychobiological factors. In particular, multiple measurements of endocrine parameters over time would be preferable in order to account for intraindividual hormonal fluctuation, which is especially relevant in the investigation of sex hormones in pre- and perimenopausal women. Furthermore, the use of saliva and DBS samples to measure sex hormones in middle-aged and elderly women needs to be carefully validated in future studies. Although the quantification of the mentioned hormones in saliva and DBS samples is a reliable approach and has several advantages, it may also be inferior to measuring serum levels in some samples or subgroups like postmenopausal women (see Tivis et al., 2005). Another limitation refers to the assessment of sexual functioning and the associated sample size reduction in our study. The FSFI (Rosen et al., 2000) is considered to be the gold standard in the assessment of female sexual functioning and is broadly used in clinical and epidemiological research. Due to its dependence on sexual activity, women with no engagement in sexual activity over the last 4 weeks had to be excluded from the study. According to Rosen et al. (2000), the FSFI should only be applied to women who have had sexual activity during the measurement period. From a total of 130 women participating in the Women 40+ Healthy Aging Study, 13 participants reported not having been sexually active in this time frame. Unfortunately, 14 more study participants showed inconsistencies when reporting whether they engaged in sexual activity in the last month and therefore also had to be excluded. For most of the FSFI items, it was possible to select *“no sexual activity”* as a response option, leading to within-subject inconsistencies across different items in terms of the reported sexual (in-)activity in these women. We can only speculate about possible reasons for this response behavior. We did not find any differences between the women who responded inconsistently and the rest of the sample with regard to health variables or relationship status. However, it is interesting to note that for several of the women concerned, sexual activity was reported primarily at the beginning of the questionnaire and sexual inactivity toward the end. This may be due to the occurrence of partner-related questions toward the end (e.g., item number 14: “Over the past 4 weeks, how satisfied have you been with the amount of emotional closeness during sexual activity between you and your partner?”). Possible explanations are that some of the women did not want to answer sexual partner-related questions or that the instructions of the questionnaire regarding the definition of sexual activity had not been understood correctly. Alternatively, the inconsistent response behavior may be due to a potential unwillingness to answer some of the questions for reasons of privacy or non-disclosure. Thus, our results are important in that they demonstrate that sexuality is still a rather taboo topic that may be regarded with shame in some middle-aged and older women.

Some practical implications may be derived from our findings. The relevance of psychosocial factors such as social support or self-esteem for female sexuality in midlife and older age suggests potential starting points from which to foster sexual functioning. Psychotherapeutic strategies targeting the social context, self-concept or aspects of personality may help to optimize women’s sexual functioning in aging. Furthermore, in clinical practice, the complete assessment of biopsychosocial aspects contributing to sexual functioning is necessary in order to guide the management of middle-aged and older adult’s sexual health.

In conclusion, this study showed that sexual functioning in healthy middle-aged and older women is highly dependent on psychosocial aspects that are health-promoting or related to well-being, such as interpersonal factors and protective psychological traits. Endocrine factors seem to be of secondary importance in this regard. To further investigate female sexual health and its associated psychobiological underpinnings, a focus on sexual function rather than dysfunction may be the better approach.

## Author Contributions

All authors conceived and planned the study, contributed to the interpretation of the results, provided critical feedback, and helped shape the research, analysis and manuscript. LM took the lead in writing the manuscript.

## Conflict of Interest Statement

The authors declare that the research was conducted in the absence of any commercial or financial relationships that could be construed as a potential conflict of interest.
